# Identification of miR‐30c‐5p as a tumor suppressor by targeting the m^6^A reader HNRNPA2B1 in ovarian cancer

**DOI:** 10.1002/cam4.5246

**Published:** 2022-10-18

**Authors:** Qiulei Wu, Guoqing Li, Lanqing Gong, Jing Cai, Le Chen, Xiaohan Xu, Xiaoli Liu, Jing Zhao, Ya Zeng, Rui Gao, Lili Yu, Zehua Wang

**Affiliations:** ^1^ Department of Obstetrics and Gynecology, Union Hospital, Tongji Medical College Huazhong University of Science and Technology Wuhan China

**Keywords:** Biological function, HNRNPA2B1, m^6^A level, miR‐30c‐5p, ovarian cancer

## Abstract

**Background:**

microRNAs (miRNAs) and N6‐methyladenosine (m^6^A) play important roles in ovarian cancer (OvCa). However, the mechanisms by which miRNAs regulate m^6^A in OvCa have not been elucidated so far.

**Methods:**

To screen m^6^A‐related miRNAs, Pearson's correlation analysis of miRNAs and m^6^A regulators was implemented using The Cancer Genome Atlas database (TCGA). To determine the level of m^6^A, RNA m^6^A quantitative assays were used. Then, colony formation assays, EdU assays, wound healing assays, and Transwell assays were performed. The dual‐luciferase reporter assay was used to confirm the miRNA target genes. Protein–protein interaction (PPI) analysis of the target genes was performed, and hub genes were discovered using the cytoHubba/Cytoscape software. The underlying molecular mechanisms were explored by bioinformatics and RNA stability assays.

**Results:**

A total of 126 miRNAs were identified as m^6^A‐related miRNAs by Pearson's correlation analysis. Among them, the high level of miR‐30c‐5p was associated with good prognosis in OvCa patients. In vitro, the miR‐30c‐5p agomir lowered the m^6^A level and inhibited OvCa cell proliferation, migration, and invasion. The hub target genes of miR‐30c‐5p were identified as *(i) XPO1, (ii) AGO1, (iii) HNRNPA2B1*, of which m^6^A reader HNRNPA2B1 was highly expressed in OvCa tissues and related with poor prognosis. In vitro, knockdown of HNRNPA2B1 significantly reduced m^6^A level and hampered the proliferation and migration of OvCa cells. The inhibition of m^6^A reader HNRNPA2B1 attenuated the suppression of proliferation and migration and the low m^6^A level induced by the miR‐30c‐5p downregulation. Mechanistically, m^6^A reader HNRNPA2B1 might regulate CDK19 mRNA stability to alter m^6^A level.

**Conclusions:**

miR‐30c‐5p inhibits OvCa progression and reduces the m^6^A level by inhibiting m^6^A reader HNRNPA2B1, thus providing new insights into the m^6^A regulatory mechanism in OvCa.

## Graphical abstract

miR‐30c‐5p inhibits OvCa progression by inhibiting m6A reader HNRNPA2B. Meanwhile, HNRNPA2B1 might regulate CDK19 mRNA stability to alter m6A level.

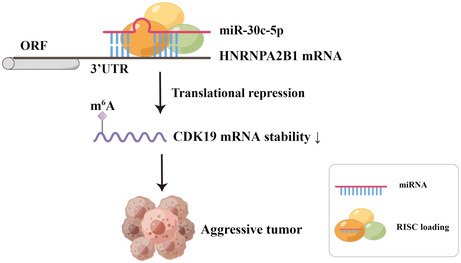

## 1 BACKGROUND

Ovarian cancer (OvCa) has been the most lethal cancer in females,^1^ and about 80% patients are identified with metastatic cancer at an advanced stage.^2^ Although surgery and platinum‐based chemotherapy respond well to initial treatment, the recurrence rate remains high for advanced patients. Thus, more research into the underlying molecular mechanism of tumor aggressiveness is critically needed to improve treatment strategies.

Epigenetics refers to gene expression and cell phenotypic changes that do not alter the DNA sequence.^3^ Recent studies indicate that epigenetic modification is potential to improve tumor treatment therapies.^4^, ^5^ Over the past few decades, studies on epigenetic modifications have focused on genomic modifications, including DNA methylation, and histone acetylation. Researchers have discovered in recent years that widespread RNA modifications in organisms serve important roles in tumorigenesis and development.^6^ N6‐methyladenosine (m^6^A) is the most frequent RNA modification, occurring in almost all phases of RNA life.^7^, ^8^ The m^6^A modification supports embryonic development and sustains hematopoietic stem cell function and other normal cellular functions by delicate balance.^9^, ^10^ The disturbance of this balance leads to a series of pathophysiological alterations, resulting in various human diseases, such as aging,^6^ metabolic disorders,^11^ cardiomyopathy,^12^ and cancers.^13^ The m^6^A methyltransferase complex (METTL3, METTL14, and WTAP) assembles the m^6^A modification, which is then erased by demethylase (FTO and ALKBH5) and identified by a collection of RNA‐binding proteins (HNRNPs, YTHDF1/2/3, IGF2BPs, and RBMX), also called as “writers”, “erasers”, and “readers”, respectively.^14^ These m^6^A regulators affect the RNA stability, which leads to OvCa malignancy and poor prognosis.^15^ Recently, drugs targeting m^6^A modification have been developed to treat cancer.^16^ The identification of critical molecules that alter m^6^A modification is significant for improving the treatment of OvCa patients.

microRNA (miRNAs), a set of evolutionarily conserved noncoding RNAs (ncRNAs), regulate gene expression at the post‐transcription level.^17^, ^18^ Recent research has shown that m^6^A modifications are linked to ncRNAs, especially miRNAs.^19^ On the one hand, m^6^A regulators execute m^6^A‐dependent modification to promote the maturity of miRNAs involved in carcinogenesis.^20^ On the other hand, miRNAs target m^6^A regulators to alter the m^6^A modification profiles to influence cancer development.^21^, ^22^, ^23^ However, the mechanisms by which miRNAs regulate m^6^A to manifest malignant behaviors of OvCa are less known.

In this finding, we identified that miR‐30c‐5p reduced the m^6^A level and limited cell growth and motility abilities by targeting the m^6^A reader HNRNPA2B1 in OvCa. Our findings reveal a new molecular mechanism for altering m^6^A level and identify miR‐30c‐5p as a potential therapeutic target for OvCa.

## 2 METHODS

### 2.1 Clinical samples

Sixty‐four tumor tissues were obtained from OvCa patients after surgical resection. Twenty‐two normal fallopian tubal and ovarian tissues were collected from patients who received a total hysterectomy and bilateral salpingo‐oophorectomy with benign gynecological diseases for use as controls in this study. All clinical samples were prepared into paraffin‐embedded sections. Written consent was obtained before surgery. The study protocol was approved by the Ethics Committee. Table 1 summarizes the clinical information of the patients.

**TABLE 1:** Clinicopathological characteristics of patients (*N* = 64)

Variables	*N*	Score of m^6^A (Mean ± SD)	*p* value
Age (years)			
<50	25	3.815 ± 2.296	0.268
≥50	39	4.496 ± 2.497	
FIGO stage			
Stages I–II	18	3.389 ± 1.771	0.369
Stages III–IV	46	4.235 ± 2.531	
Histological Type			
HGSOC	42	3.473 ± 1.744	0.209
Non‐HGSOC	22	4.271 ± 2.602	
Omentum metastasis			
No	29	3.234 ± 2.054	0.017
Yes	35	4.629 ± 2.436	

Abbreviations: FIGO, international federation of gynecology and obstetrics; HGSOC, high‐grade serous ovarian cancer.

### 2.2 Immunohistochemistry (IHC) staining

After dewaxing and rehydrating paraffin sections, antigen retrieval was carried out in sodium‐citrate buffer at 95°C. The endogenous peroxidase was then inactivated with 3% H_2_O_2_, and nonspecific binding sites were blocked with 10% goat serum. The sections were incubated with primary antibodies against m^6^A (1:200, Abcam, ab151230) at 4°C overnight and cultured with the biotin‐conjugated IgG the next day. The sections were then stained with hematoxylin and 3,3'diaminobenzidine (DAB). By multiplying the intensity score by the percentage staining area, the total score was calculated (total from 0 to 9).

### 2.3 Source of datasets

The Cancer Genome Atlas (TCGA; http://tcga‐data.nci.nih.gov/tcga) database was used to identify m^6^A‐related miRNAs in OvCa. The expression datasets of the normal tissues were obtained from the Genotype‐Tissue Expression (GTEx; https://gtexportal.org/home/) database. Other OvCa datasets, including GSE101976, GSE119055, GSE83693, GSE27651, GSE66957, and GSE27651, were downloaded from the Gene Expression Omnibus (GEO; www.ncbi.nlm.nih.gov/geo/) database.

### 2.4 Cell culture and transfection

Human OvCa cell lines (SKOV3, ES2, A2780, CAOV3, OVCAR3, and OVCAR4) were obtained from the China Center for Type Culture Collection (Wuhan University). OVCAR3 cells were cultivated in RPMI‐1640 medium containing 20% fetal bovine serum (FBS, Gibco), whereas SKOV3, ES2, A2780, CAOV3, and OVCAR4 cells were cultured in DMEM/F12 medium supplemented with 10% FBS in a humidified incubator (37°C, 5% CO_2_). The HNRNPA2B1 small interfering RNAs (siRNAs) and miR‐30c‐5p agomirs/antagomirs were obtained from RiboBio. Lipofectamine 3000 (Invitrogen) was used for transfection assays, which were performed according to the manufacturer's instructions. The corresponding sequences are listed in Table S1.

### 2.5 Quantitative real‐time PCR (qRT‐PCR)

Total RNA from OvCa cells was isolated using TRIzol reagent (Takara). The HiScript III qRT SuperMix Kit (Vazyme) and Prime‐Script RT Master Mix Kit (Takara) were used to reverse transcription, and the relative level of each RNA was determined using SYBR Green (Vazyme). Table S1 listed the corresponding primer sequences. Each experiment has three replicates.

### 2.6 RNA m^6^A quantitative assay

The EpiQuik m^6^A RNA Methylation Quantification Kit (Epigentek) was used to measure the m^6^A level of total RNAs. The m^6^A level was quantified by reading the absorbance at 450 nm using a Spectramax plate reader (SpectraMax i3). Each experiment has three replicates.

### 2.7 Transwell assay

Forty‐eight hours after transfection, 4 × 10^4^ cells in serum‐free medium were seeded into the upper chamber without (Transwell migration assay) or with (Transwell invasion assay) Matrigel (BD Biosciences). After 24 h of incubation, non‐migrated or invaded cells were scraped off using a cotton swab, and cells on the bottom of the chamber were fixed with methanol for 10 min and stained using 0.1% crystal violet. Then, five fields were selected and photographed randomly. Each experiment was repeated three times.

### 2.8 Wound healing assay

OvCa cells formed a confluent monolayer in six‐well plates, wounded with a 200‐μl pipette tip. After replacing the culture media with serum‐free medium, the wound was closed after 24 h. Each experiment has three replicates. The wound healing areas were observed by and measured by ImageJ software (version 1.51).

### 2.9 5‐Ethynyl‐2′‐Deoxyuridine (EdU) cell proliferation assay

According to the manual (RiboBio) to perform the assays, all images were taken with an Olympus fluorescence microscope of five random fields. All experiments have three replicates at least.

### 2.10 Colony formation assay

Five hundred cells per well were plated in 6‐well plates and cultured until colonies were visible. After being fixed with 4% formaldehyde, the colonies were treated with 0.1% crystal violet. All experiments have three replicates at least.

### 2.11 miRNA target prediction and protein–protein interaction (PPI) network

Online prediction tools included miRWalk databases (http://mirwalk.umm.uni‐heidelberg.de/), miRDB databases (http://mirdb.org/), miRTarBase databases (http://mirtarbase.mbc.nctu.edu.tw/index.html), and TargetScan databases (http://www.targetscan.org/vert_80/). The online websites Kaplan–Meier plotter (KM plotter) and UALCAN were used to determine the prognostic significance and the expression of genes.^24^ In addition, the interaction among the genes was analyzed by the STRING database (https://string‐db.org/). The Hub genes were analyzed by the Molecular Complex Detection (MCODE) plugin in Cytoscape (version 3.8.2). (degree cut‐off = 2, node score cut‐off = 0.2, k‐core = 2, and max. Depth = 100).

### 2.12 Functional enrichment analysis

The clusterProfiler package was used to reveal the functions and pathways of miR‐30c‐5p target genes using Gene Ontology (GO) and Kyoto Encyclopedia of Genes and Genomes (KEGG) enrichment. In addition, miRNACancerMAP was utilized to investigate the miR‐30c‐5p cancer pathways.^25^

### 2.13 HNRNPA2B1 immunostaining

For immunocytofluorescence (ICF) assays, the cells were fixed with 4% formaldehyde. After incubating with 0.1% Triton X‐100 and 3% bovine serum albumin (BSA), the cells were incubated with anti‐HNRNPA2B1 antibody (1:400, Proteintech, 14,813‐1‐AP) overnight at 4°C. Then, Cy3–conjugated IgG and FITC phalloidin (Yeasen) were used to stain the cells, followed by DAPI staining. For immunocytochemistry (ICC) assays, the cells were treated with biotin‐conjugated IgG at 37°C and stained with DAB, hematoxylin, and eosin. A fluorescent microscope was used to image the cell morphology (Olympus). All experiments have three replicates at least.

### 2.14 Western blotting

RIPA buffer was used to extract total cellular protein. Protein extracts were separated in 10% SDS‐PAGE and transferred to PVDF membranes. Membranes were blocked with 5% defatted milk. After incubation with the primary antibodies at 4°C overnight, the membrane was treated with HRP‐conjugated secondary antibody (1:5000, CST). Protein bands were detected using an advanced chemiluminescence kit (Pierce, ThermoScientific) in Molecular Imager ChemiDoc XRS+ and Image Lab software. The primary antibodies used were anti‐HNRNPA2B1 (1:8000, Proteintech, 14,813‐1‐AP) and anti‐β‐actin (1:5000, Proteintech, 66,009‐1‐Ig). All experiments were repeated thrice at least.

### 2.15 Luciferase reporter gene assay

The wild‐type or mutated sequence of the *HNRNPA2B1* mRNA 3'UTR was cloned into pmirGLO vector. Cells were co‐transfected with the specific luciferase reporter plasmids. The assays were detected by the Dual‐Luciferase Reporter Assay (E1910, Promega). Each experiment was repeated at least three times.

### 2.16 Prediction of RNA‐protein interaction and m^6^A site

The potential interaction of HNRNPA2B1 and miR‐30c‐5p was assessed by use of the RNA–protein interaction prediction tool (RPISeq), which is based on random forest (RF) or support vector machine (SVM). Predictions with probabilities >0.5 were considered positive. The online URL: http://pridb.gdcb.iastate.edu/RPISeq/references.php. The potential m^6^A sites were predicted using an online tool, SRAMP (http://www.cuilab.cn/sramp/).

### 2.17 RNA stability analysis

The cells were treated with mRNA transcription inhibitor actinomycin D (5 μg/ml) (MCE, HY‐17559) for 0, 2, 4, and 6 h. Cells were collected, and the total RNA was extracted by TRIzol Reagent (Invitrogen) and analyzed by qPCR. Each experiment was repeated at least three times.

### 2.18 Statistical analysis

Statistical analyses were carried out by using R Statistical Software (version 3.6.3) and GraphPad Prism (version 8.0.1). All data are expressed as mean ± standard deviation (mean ± SD). Pearson's correlation analysis was used to measure correlation. Student's *t* test or one‐way ANOVA was used to evaluate the differences between two or multiple groups, respectively. The Mann–Whitney test and Kruskal‐Wallis test were used for nonnormally distributed data. Statistical significance was set at *p* < 0.05.

## 3 RESULTS

### 3.1 Dysregulation of m^6^A level and m^6^A regulators in OvCa

**FIGURE 1:**
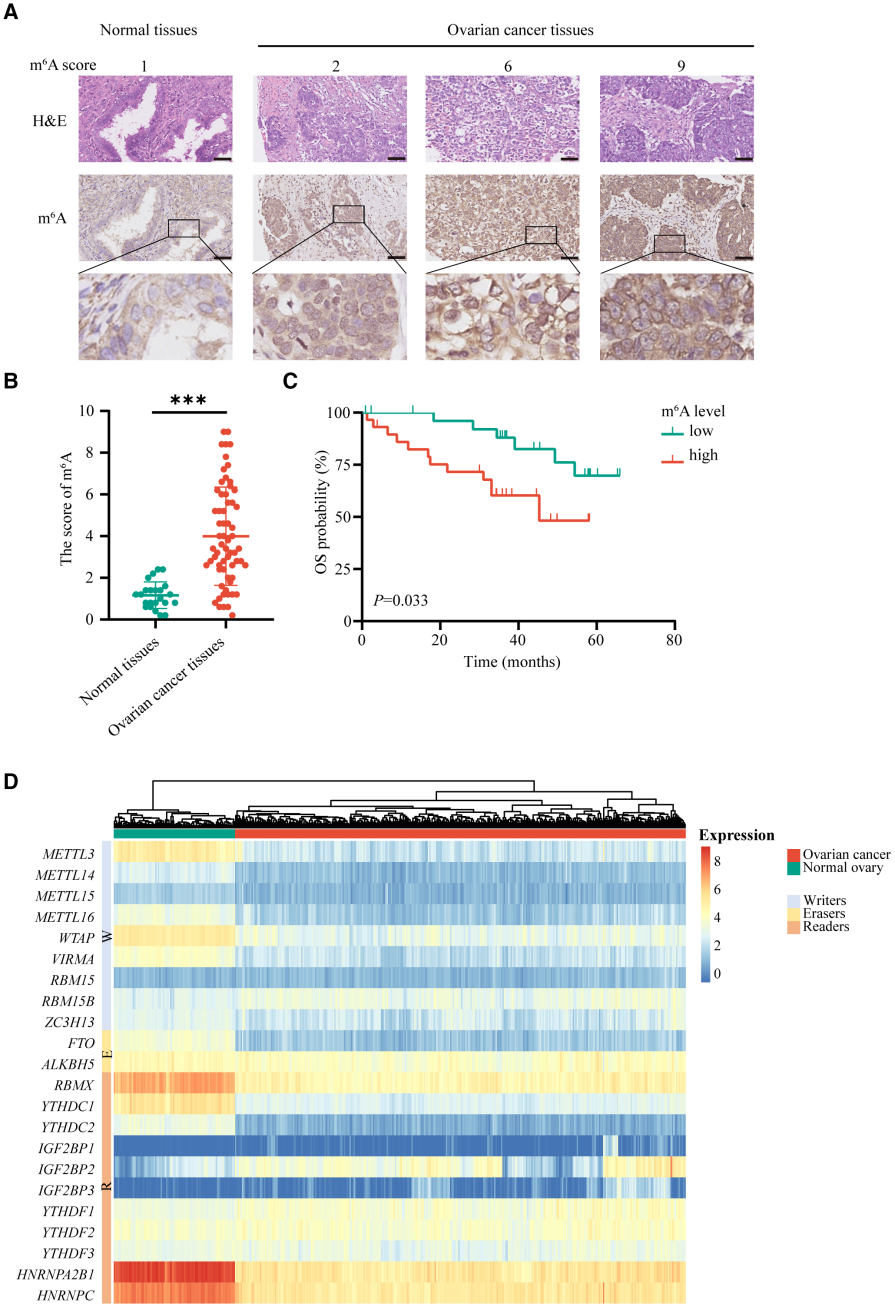
Disregulation of m^6^A level and m^6^A regulators in OvCa. (A) Representative IHC images of m^6^A in tissues. Scale bar 50 μm. (B) m^6^A level in normal tissues and OvCa tissues. (C) Kaplan–Meier analysis of m^6^A level in OvCa patients. (D) Heatmap for the mRNA levels of 22 m^6^A regulators. *p < 0.05; **p < 0.01; ***p < 0.001; ns. not significant

Analysis of the IHC revealed that the m^6^A level was markedly upregulated in OvCa tissues than in normal controls (Figure 1A,B and Figure S1). Moreover, the overall survival (OS) of patients with high m^6^A level exhibited was shorter than of those in the low group (Figure 1C). These results suggest that m^6^A modification involves in the progression of OvCa.

Considering that m^6^A modification is mediated by m^6^A regulators, we identified the levels of the 22 m^6^A regulators between OvCa tissues and normal controls using datasets from the TCGA and GTEx databases. The findings revealed that the 22 m^6^A regulators were expressed variably, indicating that the m^6^A alternation might participate in OvCa tumorigenesis and development (Figure 1D and Figure S2A).

### 3.2 Identification of m^6^A‐related miRNAs in OvCa

**FIGURE 2:**
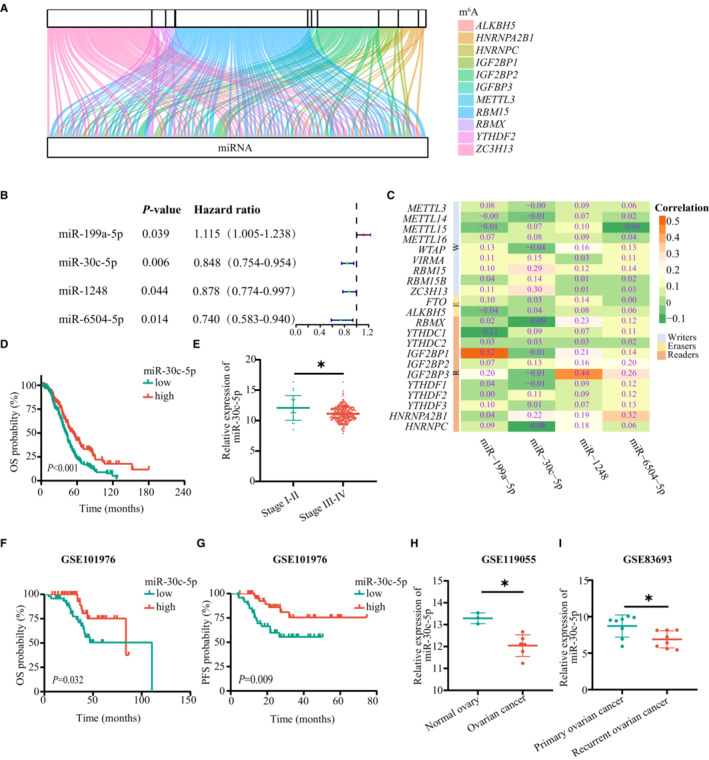
Identification of m^6^A‐related miRNAs in OvCa. (A) Sankey relational diagram for 22 m^6^A regulators and miRNAs. (B) Forest plot for the OS‐associated miRNAs. (C) Heatmap for the correlations between 22 m^6^A regulators and the four prognostic m^6^A‐related miRNAs. (D) Level of miR‐30c‐5p in OvCa in different FIGO stages in the TCGA database. (E) Kaplan–Meier OS curve for OvCa patients in the TCGA database. Kaplan–Meier analysis of OvCa patients in GSE101976 of OS (F) and PFS (G) Level of miR‐30c‐5p for OvCa patients in GSE119055 (H) and GSE83693 (I). *p < 0.05; **p < 0.01; ***p < 0.001; ns. not significant

To identify m^6^A‐related miRNAs in OvCa, the TCGA database was used to identify the matrix expression of 22 m^6^A regulators and 2155 miRNAs. Then, we defined m^6^A‐related miRNAs as miRNAs that had a strong relationship with one or more of the 22 m^6^A regulators. Pearson's analysis was performed to determine the correlation (|Pearson *R*| > 0.3 and *p* < 0.05), and the m^6^A regulators‐miRNAs co‐expression network was visualized by a Sankey diagram (Figure 2A). A total of 126 miRNAs were identified as m^6^A‐related miRNAs, and univariate Cox regression analysis was implemented to distinguish prognostic‐related miRNAs. As shown in Figure 2B, miR‐30c‐5p, miR‐1248, miR‐199a‐5p, and miR‐6504‐5p were significantly related to the OS of OvCa patients. The correlation between 22 key m^6^A regulators and four OS‐associated miRNAs in TCGA is shown in Figure 2C.

To single out the key m^6^A‐related miRNAs in OvCa, survival analyses from the TCGA database were performed on the four miRNAs selected above. miR‐30c‐5p (*p* < 0.001) and miR‐1248 (*p* = 0.004) were associated with OS, but miR‐199a‐5p (*p* = 0.169) and miR‐6504‐5p (*p* = 0.088) were not (Figure 2D and Figure S2B). The GSE101976 dataset showed that the OvCa patients with high level of miR‐30c‐5p had longer OS and progression‐free survival (PFS), which is consistent with the TCGA database. Considering that the International Federation of Gynecology and Obstetrics (FIGO) staging of OvCa is closely related to prognosis, the level of miRNAs in different stages was analyzed, revealing that miR‐30c‐5p expression was decreased in patients with advanced‐stage disease (FIGO stages III–IV) compared with early‐stage disease (FIGO stages I–II); however, this was not the case for miR‐1248 (Figure 2E and Figure S2C). The GSE119055 and GSE83693 datasets validated our results, revealing that the expression of miR‐30c‐5p is decreased in OvCa tissues compared with normal controls, especially in recurrent OvCa lesions (Figure 2H,I). These findings suggest that miR‐30c‐5p, an m^6^A‐related miRNA, inhibits the progression of OvCa.

### 3.3 miR‐30c‐5p decreases m^6^A level and inhibits OvCa cells proliferation, migration and invasion in vitro

**FIGURE 3:**
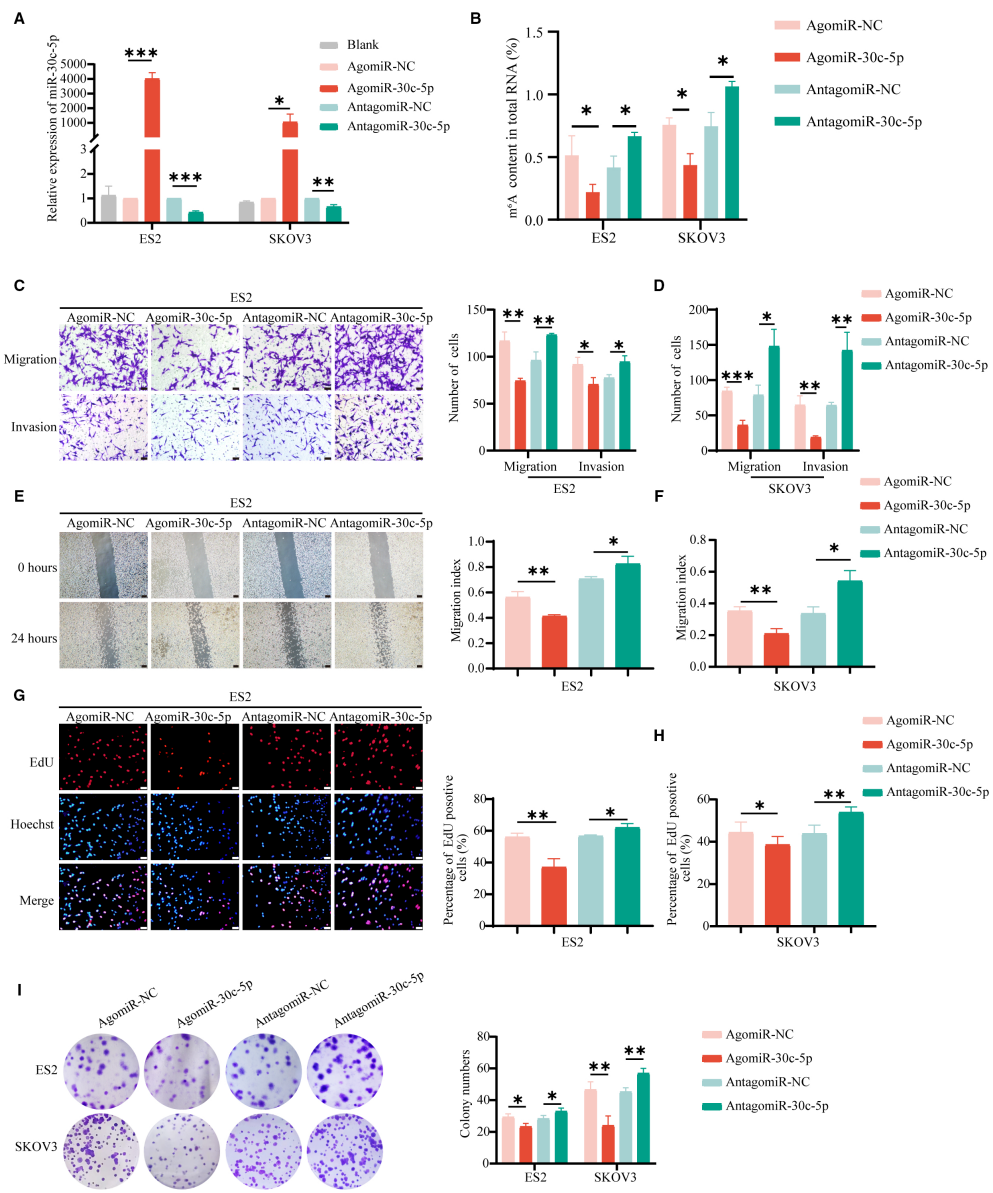
miR‐30c‐5p decreases m^6^A level and inhibits OvCa cells progression in vitro. (A) RT‐qPCR for the interference efficiencies of agomiR‐30c‐5p and antagomiR‐30c‐5p. (B) m^6^A level of ES2 and SKOV3 cells transfected with agomiR‐30c‐5p and antagomiR‐30c‐5p. Transwell assays (C, D), wound healing assays (E, F), EdU assays (G, H), and colony formation assays (I) of ES2 and SKOV3 cells transfected with agomiR‐30c‐5p and antagomiR‐30c‐5p. Wound healing scale bar 200 μm. EdU assays and Transwell assays scale bar 20 μm. Each experiment has three replicates at least. *p < 0.05; **p < 0.01; ***p < 0.001; ns. not significant

To investigate how miR‐30c‐5p regulates the biological function in vitro, we transfected miR‐30c‐5p agomir (an agonist) and miR‐30c‐5p antagomir (an inhibitor) into OvCa cells and performed the qRT‐PCR assays to validate the transfection efficiency (Figure 3A). The RNA m^6^A quantitative experiment showed that the m^6^A level in the agomiR‐30c‐5p group was lower than that of the negative control (NC) group and vice versa (Figure 3B). In SKOV3 and ES2 cells, overexpression of miR‐30c‐5p reduced motility and invasion (Figure 5C‐F). Meanwhile, the miR‐30c‐5p agomir markedly reduced the number of clones and EdU‐positive proliferating cells (Figure 5G‐I). Accordingly, the migrative, invasive, and proliferative abilities of OvCa cells increased after downregulation of miR‐30c‐5p. These results suggested that miR‐30c‐5p could decrease m^6^A level and inhibit the progression of OvCa in vitro.

### 3.4 miR‐30c‐5p targets m^6^A reader *HNRNPA2B1*

**FIGURE 4:**
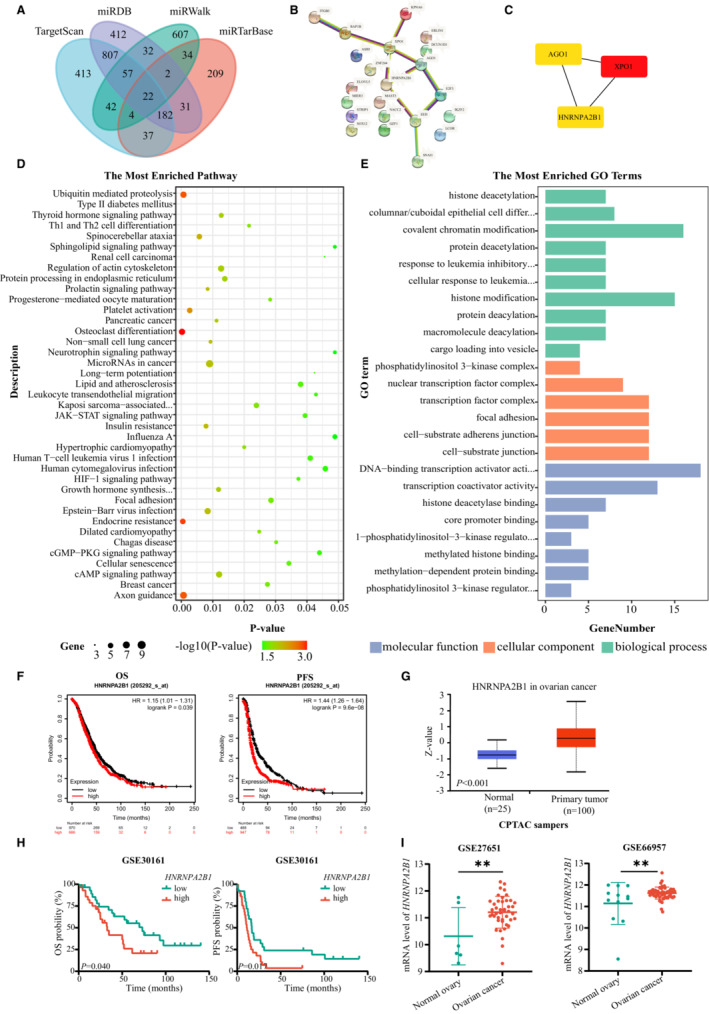
Hub target genes of miR‐30c‐5p. (A) Venn diagram of miR‐30c‐5p target genes. PPI networks (B) and three hub genes (C) of 22 overlapping genes. KEGG (D) and GO enrichment analysis (E) of the 22 overlapping genes. (F) Kaplan–Meier OS and PFS curve of HNRNPA2B1 in OvCa patients. (G) Protein expressions of the HNRNPA2B1. (H) Kaplan–Meier analysis of OvCa patients in GSE30161 of OS and PFS. (I) Different mRNA levels of the HNRNPA2B1 between normal and tumor tissue in GSE27651 and GSE66957. *p < 0.05; **p < 0.01; ***p < 0.001; ns. not significant

Four online miRNA target analysis websites (TargetScan, miTarBase, miRDB, and miWALK) were used to predict the potential target genes, and the 22 overlapping genes were recognized (Figure 4A). PPI networks of the 22 overlapping genes were then visualized in CytoScape, and the top three hub genes (*XPO1, AGO1*, and *HNRNPA2B1*) were clustered using MCODE (Figure 4B,C). As presented in Figure 4D,E, GO and KEGG enrichment analyses of the 22 overlapping genes were mainly enriched in the transcription and posttranscriptional modification and the JAK–STAT/HIF‐1 signaling pathway. Moreover, the miRNACancerMAP online website showed that miR‐30c‐5p is closely related to the tumor‐associated signaling pathways, including the AKT pathway and ErbB pathway (Figure S3A). These target genes appeared to play important roles in OvCa carcinogenesis by regulating cancer cell proliferation and motility. Hub genes are highly connected genes in gene expression networks and are inclined to play important roles in biological mechanisms. Further studies were performed to investigate the prognostic significance of the three hub genes. Kaplan–Meier survival curves and log‐rank test analyses indicated that patients with high HNRNPA2B1 protein expression were related to decreased PFS and OS (Figure 4F). Meanwhile, HNRNPA2B1 expressed more highly in OvCa lesions than in normal tissues (Figure 4G), which was consistent with the results from GSE23554, GSE27651, and GSE66957 (Figure 4H,I), while XPO1 and AGO1 had the opposite expression and prognosis pattern (Figure S3B‐E). In general, m^6^A reader HNRNPA2B1 plays oncogenic roles in OvCa.

**FIGURE 5:**
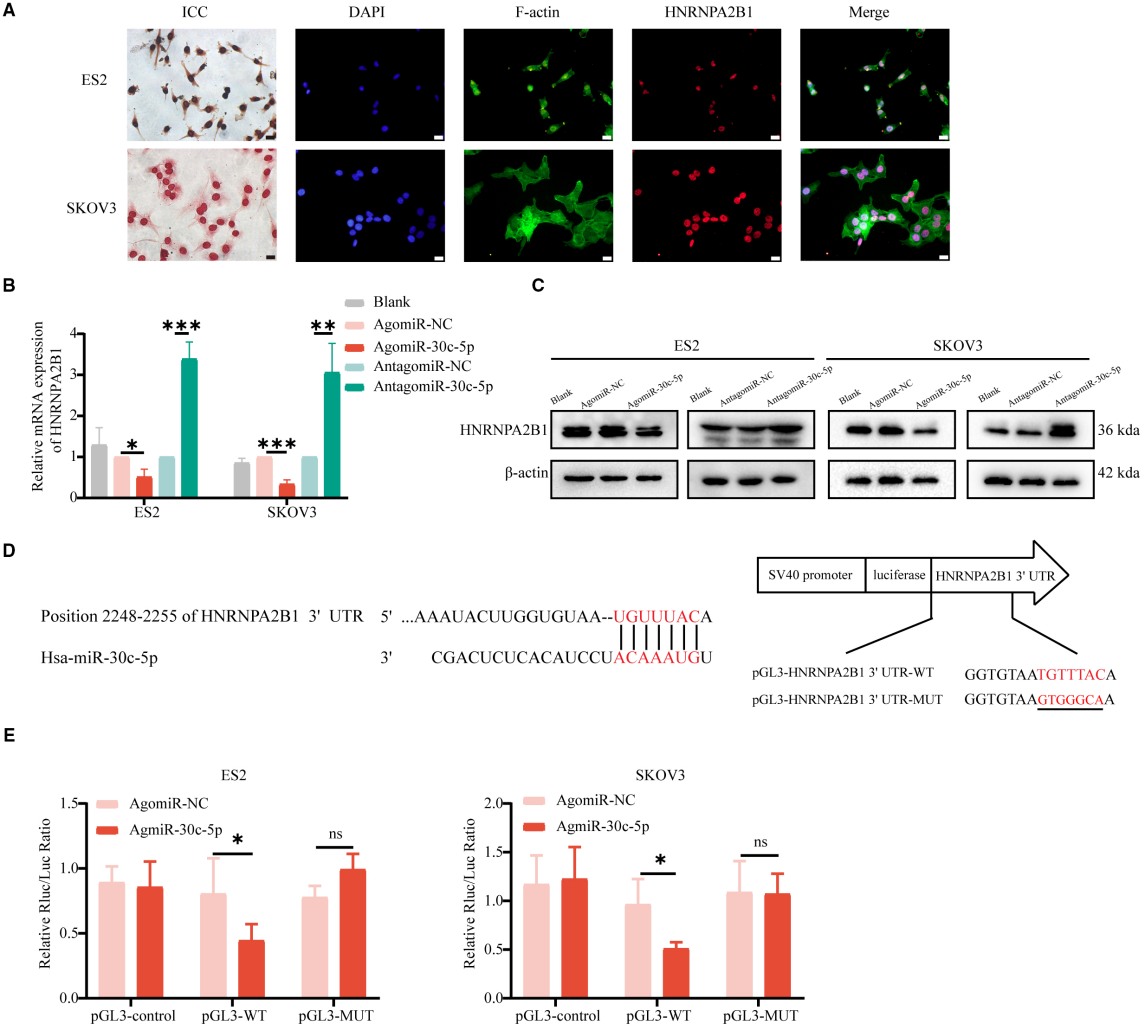
miR‐30c‐5p targets m^6^A reader *HNRNPA2B1*. (A) Representative ICC and ICF images of HNRNPA2B1 in SKOV3 and ES2 cells. Scale bar 20 μm. (B) qRT‐PCR for the mRNA level of HNRNPA2B1. (C) Representative images of Western blotting for the HNRNPA2B1 protein. (D) Schematic diagram for the sequences of wild‐type and mutation of HNRNPA2B1. (E) Dual‐luciferase reporter assay in SKOV3 and ES2 cells. Each experiment has three replicates at least. *p < 0.05; **p < 0.01; ***p < 0.001; ns. not significant

Considering that the protein function is determined by its location, ICF and ICC assays were performed and revealed the nuclear location of HNRNPA2B1 in different OvCa cell lines (Figure 5A and Figure S4A). And, qRT‐PCR assays and Western blotting assays were performed and revealed that miR‐30c‐5p agomir reduced HNRNPA2B1 expression and vice versa (Figure 5B,C). After validating transfection efficiency (Figure S4B), similar results were validated in other OvCa cells, including A2780, CAOV3, OVCAR3, and OVCAR4 cells (Figure S4C). Then, dual‐luciferase reporter assays showed that miR‐30c‐5p significantly reduced the luciferase activity of the wild‐type group of *HNRNPA2B1*, but no significant reduction was observed in the *HNRNPA2B1* mutation group (Figure 5D,E). In conclusion, miR‐30c‐5p targets m^6^A reader *HNRNPA2B1*.

### 3.5 miR‐30c‐5p inhibits m^6^A level and OvCa cells proliferation, migration and invasion by targeting HNRNPA2B1 in vitro

**FIGURE 6:**
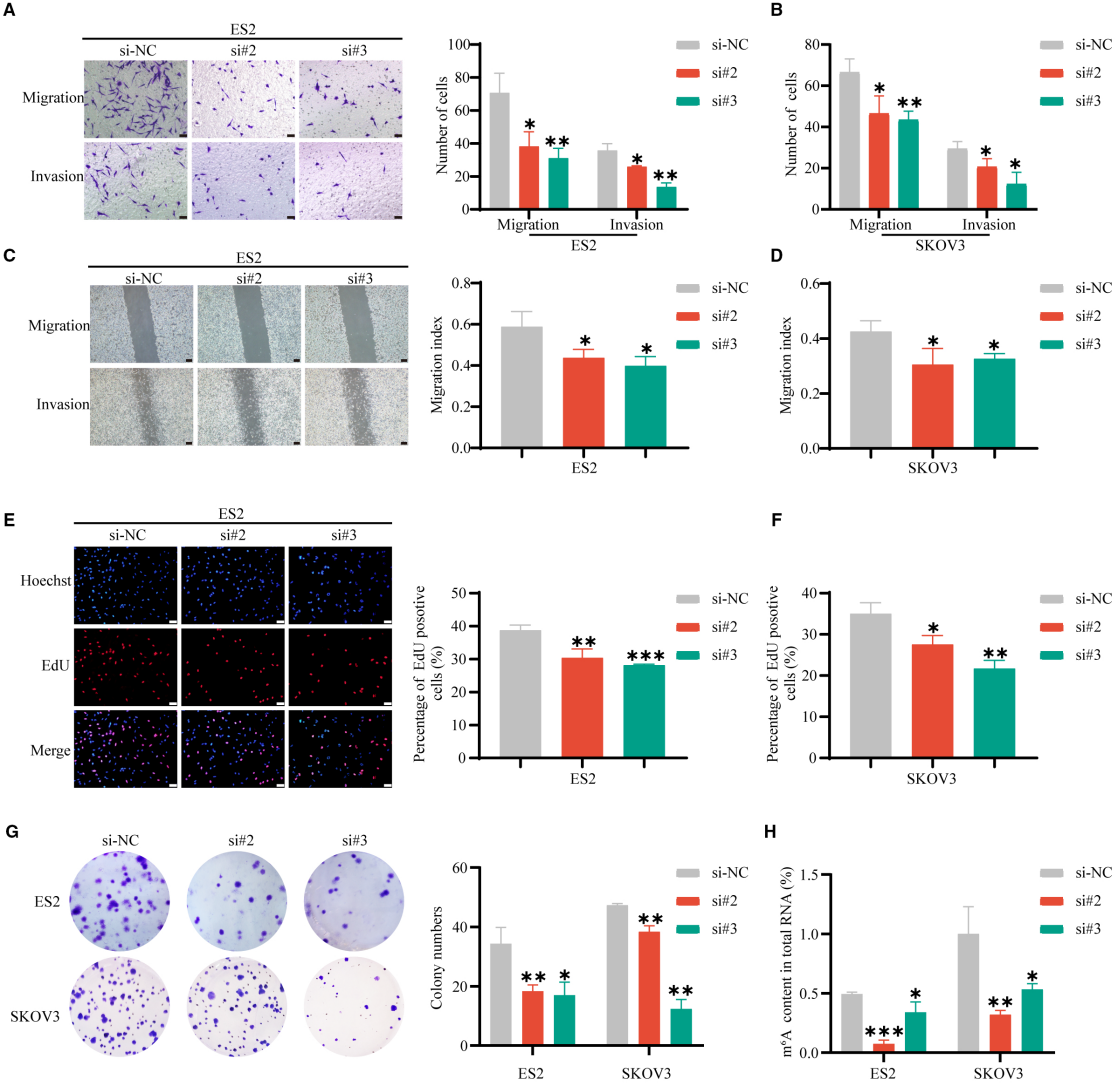
miR‐30c‐5p inhibits m^6^A level and OvCa cells progression by targeting HNRNPA2B1 in vitro. Transwell assays (A, B), wound healing assays (C, D), EdU assays (E, F), and colony formation assays (G) of ES2 and SKOV3 cells after downregulation of HNRNPA2B1. (H) m^6^A level of ES2 and SKOV3 cells after downregulation of HNRNPA2B1. Wound healing scale bar 200 μm. EdU assays and Transwell assays scale bar 20 μm. Each experiment has three replicates at least. *p < 0.05; **p < 0.01; ***p < 0.001; ns. not significant

To further study the biological function of HNRNPA2B1 in OvCa in vitro, small interfering RNAs (siRNAs) were used to decrease m^6^A reader HNRNPA2B1 expression. The silencing efficiency was validated by qRT‐PCR and Western blotting assays (Figure S4D,E), and si#2 and si#3 were chosen to conduct the following experiments because of their relatively high silencing efficiency. Transwell migration assays and wound‐healing assays showed that the number of migrated ES2 and SKOV3 cells significantly decreased after HNRNPA2B1. Moreover, Transwell invasion assays demonstrated that HNRNPA2B1 knockdown significantly reduced the invasion abilities (Figure 6A‐D). Thus, HNRNPA2B1 knockdown inhibited the migration and invasion capacity of OvCa cells. EdU assays and cloning formation assays showed that the proliferation ability of OvCa cells, including ES2 and SKOV3 cells, decreased after knocking down HNRNPA2B1 (Figure 6E‐G). The RNA m^6^A quantitative experiments were performed and showed that downregulation of HNRNPA2B1 reduced the m^6^A level (Figure 6H).

**FIGURE 7:**
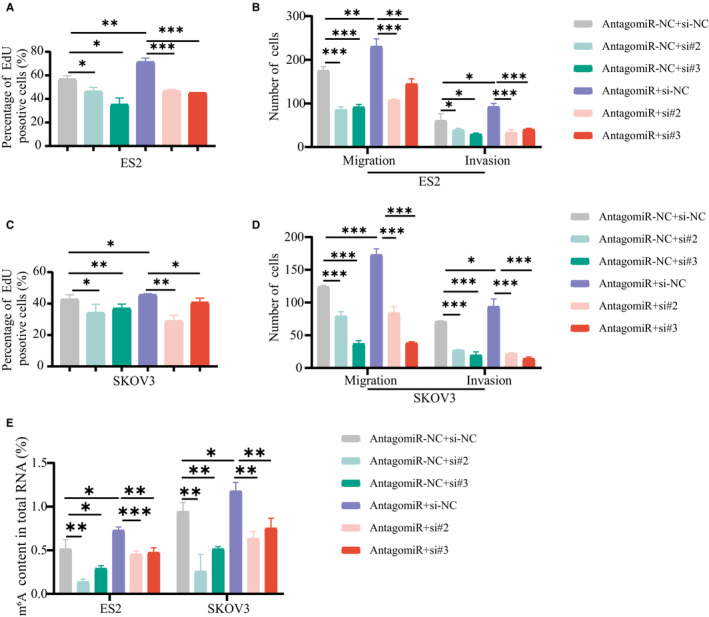
Downregulation of HNRNPA2B1 offsets the oncogenic activities of OvCa cells transfected with antagomiR‐30c‐5p. Edu assays (A, C) and Transwell assays (B, D) showed that inhibition of miR‐30c‐5p reversed the migration, invasion, and proliferation ability repressed by downregulation of HNRNPA2B1. (E) m^6^A level of ES2 and SKOV3 cells transfected with si‐HNRNPA2B1 and antagomiR‐30c‐5p. Each experiment has three replicates at least. *p < 0.05; **p < 0.01; ***p < 0.001; ns. not significant

To further confirm that the function of miR‐30c‐5p in OvCa progression is dependent on HNRNPA2B1, EdU assays were performed and demonstrated that the miR‐30c‐5p antagomir increased the proliferation of OvCa cells (Figure 7A,C), which was attenuated by the downregulation of HNRNPA2B1. Transwell migration and invasion assays and m^6^A quantitative assay showed that HNRNPA2B1 downexpression rescued the cell mobility (Figure 7B,D) and the m^6^A level (Figure 7E) of miR‐30c‐5p knockdown cells. The miR‐30c‐5p antagomir increased the proliferation, migration, and invasion of OvCa cells (Figure 7A‐D) and the m^6^A level (Figure 7E), which was attenuated by the downregulation of HNRNPA2B1. Collectively, we demonstrate that miR‐30c‐5p restrains OvCa progression by inhibiting the oncogenic activity of HNRNPA2B1 and the m^6^A level, suggesting that miR‐30c‐5p acts as the upstream regulator for the m^6^A modification system by targeting m^6^A reader HNRNPA2B1.

### 3.6 HNRNPA2B1 regulates the stability of *CDK19* mRNA

**FIGURE 8:**
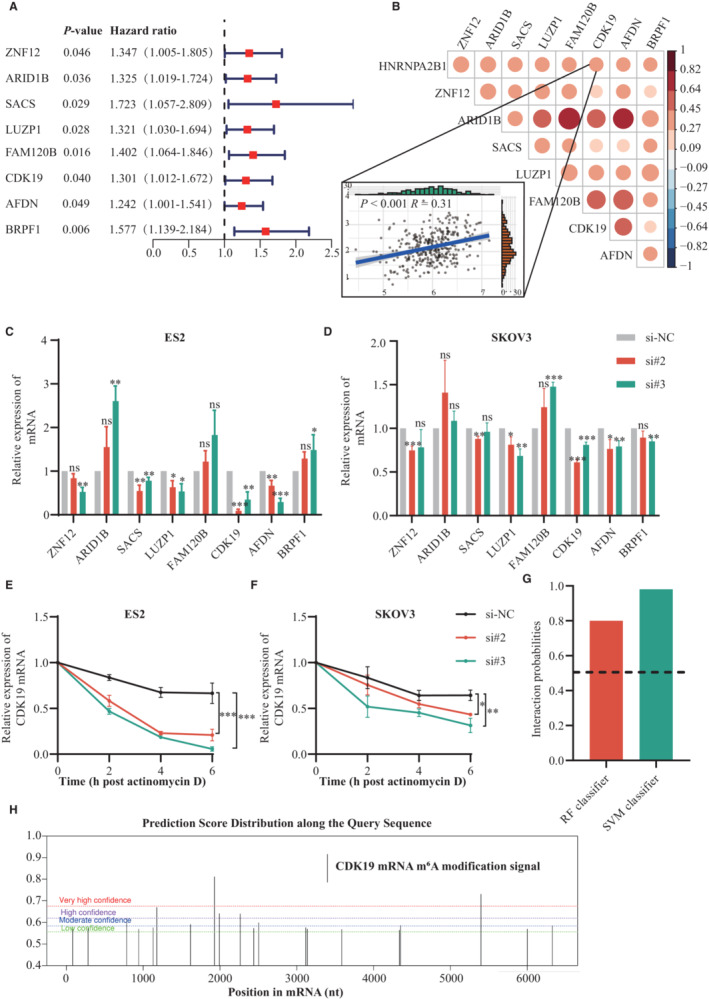
HNRNPA2B1 regulates the stability of *CDK19* mRNA. (A) Forest plot for the OS‐associated genes. (B) Pearson's correlation analysis was used to determine correlations between the eight genes. Relative expression of eight genes in ES2 (C) and SKOV3 (D) cells after downregulation of HNRNPA2B1 compared with control cells by using RT‐qPCR. Knockdown of the HNRNPA2B1 resulted in the degradation of CDK19 mRNA in ES2 (E) and SKOV3 (F) cells. (G) Prediction of the interaction probabilities of HNRNPA2B1 with CDK19 mRNA by RPISeq. (H) Potential m^6^A modification sites along mRNA of CDK19 predicted by SRAMP. Each experiment has three replicates at least. *p < 0.05; **p < 0.01; ***p < 0.001; ns. not significant

Considering that HNRNPA2B1 has an important effect on the stabilization of mRNA, Pearson's correlation analysis was used to determine the correlation between the HNRNPA2B1 and mRNA in the TCGA database (Pearson *R* > 0.3 and *p* < 0.05). Then, univariate Cox regression analysis was performed and showed that eight genes had negative effects on survival among them (Figure 8A). Co‐expression analysis of the eight genes in OvCa patients in the TCGA dataset was carried out using Pearson's correlation coefficients (Figure 8B). As shown in Figure 8C,D, qRT‐PCR results revealed that HNRNPA2B1 knockdown could significantly downregulate CDK19 expression in ES2 and SKOV3 cells, indicating that CDK19 might act as a downstream target of HNRNPA2B1. RNA stability assays further identified that the half‐life of *CDK19* mRNA was reduced in ES2 and SKOV3 cells after knockdown of HNRNPA2B1 (Figure 8E,F). As shown in Figure 8G, we demonstrated that the possible interaction between HNRNPA2B1 and *CDK19* mRNA was very high, which was assessed by RPISeq websites (RF classifier = 0.8, SVM classifier = 0.98). Meanwhile, several potential m^6^A sites were found on *CDK19* mRNA using the online tool SRAMP (Figure 8H). These data suggest that HNRNPA2B1 might bind to the m^6^A site of CDK19 mRNA to promote the stability of *CDK19* mRNA, which alters the m^6^A level of total RNA.

## 4 DISCUSSION

m^6^A modification, the most prevalent type of epigenetic regulation in cells, is an emerging field in the study of tumorigenicity of OvCa. However, the function of miRNAs involved in m^6^A regulation is still poorly understood. This study investigated how miR‐30c‐5p regulated HNRNPA2B1 to modulate OvCa progression in an m^6^A‐dependent manner. Our findings will help develop novel therapies targeting m^6^A modification in OvCa.

A series of miRNAs have been verified to regulate m^6^A modification.^21^, ^26^ In this study, we identified that four m^6^A‐related miRNAs (miR‐30c‐5p, miR‐1248, miR‐199a‐5p, and miR‐6504‐5p) were significantly related to the OS of OvCa patients. miR‐30c‐5p has been reported as a tumor suppressor in various cancers.^27^, ^28^, ^29^ Several studies also have shown that miR‐1248 and miR‐6504‐5p are related to various cancers, such as lung cancer,^30^ prostate cancer,^31^ and breast cancer.^32^ Gan, et al. demonstrated that miR‐199a‐5p targes Beclin1 and RUNX1 to regulate proliferation and invasion in OvCa.^33^ Among the four m^6^A‐related miRNAs, miR‐30c‐5p was chosen to further study because of its being significantly related to survival. Consistently, we observed that miR‐30c‐5p inhibited OvCa cell proliferation, migration, and invasion. Furthermore, we validated that miR‐30c‐5p reduced the m^6^A level in OvCa cells for the first time. These results prompt us to investigate how miR‐30c‐5p acts as a tumor suppressor to regulate the m^6^A level in OvCa.

miRNAs influence the translation or stability of mRNA by binding to the 3'UTR of mRNA. miR‐30c‐5p has been reported to target different genes to promote cancer cell proliferation and invasion.^27^, ^28^ Other studies have shown that miRNAs target m^6^A regulators to alter the m^6^A level.^21^, ^22^, ^23^ For example, miR‐501‐3p diminishes m^6^A level by targeting WTAP in kidney cancer.^23^ Sun, et al. also clarified the critical role of the miR‐103‐3p/METTL14/m^6^A signaling axis in osteoblast activity.^26^ In this study, bioinformatics approaches were utilized to predict and identify the target genes of miR‐30c‐5p, and we found that m^6^A reader HNRNPA2B1 was a novel target of miR‐30c‐5p in OvCa, as indicated by bioinformatics approaches and the luciferase assay. Further experiments proved that miR‐30c‐5p could target HNRNPA2B1 to reduce m^6^A level.

m^6^A reader HNRNPA2B1 is mainly localized in the nucleus and selectively binds to m^6^A‐containing transcripts to regulate RNA production and metabolism, including maintaining RNA stability, RNA splicing, and RNA processing.^34^, ^35^ The role of HNRNPA2B1 as an oncogene in OvCa development has been described in existing studies.^36^, ^37^, ^38^ For instance, HNNRPA2B1 facilitates the proliferation abilities of OvCa cells by regulating Lin28B expression.^39^ Wang, et al. showed that HNRNPA2B1 enhances drug sensitivity in OvCa cells.^40^ Here, we found that HNRNPA2B1 promoted the proliferation, migration, and invasion of OvCa cells, verifying its potential role as an oncogene in the OvCa progression. In general, the m^6^A level is usually regulated by the m^6^A methyltransferase complex and demethylase, while m^6^A RNA‐binding proteins (“readers”) could also alter the m^6^A level indirectly by regulating the mRNA stability.^41^, ^42^, ^43^ Lu, et al. proved that m^6^A reader IMP2 regulates m^6^A level by stabilizing the ZFAS1/OLA1 axis in colorectal cancer.^44^ Previous studies also revealed that HNRNPA2B1 stabilizes ILF3 and TCF7L2 mRNA via an m^6^A‐dependent manner.^38^, ^45^ However, whether the m^6^A reader HNRNPA2B1 regulates the mRNA stability in OvCa has not been reported so far. Herein, we demonstrated that the mRNA stability of CDK19 mRNA was significantly reduced after hnRNPA2B1 knockdown. CDK19, as a critical regulatory enzyme, has been reported to promote tumor cell migration and proliferation in prostate cancer and ovarian cancer.^46^, ^47^ Meanwhile, we found that the possibility of HNRNPA2B1 binding to CDK19 mRNA was very high, and CDK19 mRNA had several potential m^6^A modification sites by bioinformatics analysis, suggesting that HNRNPA2B1 might regulate CDK19 mRNA stability to alter m^6^A level.

miRNAs are being studied in preclinical and clinical studies in recent years, while targeted therapies for m^6^A regulators are still under investigation.^16^, ^48^, ^49^ Our results identify potential regulators of m^6^A modification and show that miR‐30c‐5p may serve as the key target to regulate m^6^A modification. Meanwhile, further animal models and experiments need to be investigated. Collectively, our study may help elucidate the processes and mechanisms of m^6^A modification in OvCa and provide novel insights into the further study of m^6^A‐related miRNAs.

## 5 CONCLUSION

This study concludes that miR‐30c‐5p reduces the m^6^A level and inhibits OvCa progression by targeting m^6^A reader HNRNPA2B1. Our findings not only identify a new potential molecular mechanism for altering m^6^A modification but also facilitate the development of novel therapies for OvCa.

## AUTHOR CONTRIBUTIONS

The study was designed by LY and ZW. QW and GL performed the experiment. LG, JC, and LC collected the clinical data. XX, XL, and JZ. YZ and RG performed bioinformatic analyses and statistical analysis.

## FUNDING INFORMATION

The National Natural Science Foundation of China (No. 81974413 and No. 81902665) provided funding for this research.

## CONFLICT OF INTEREST

No conflicts.

## ETHICS APPROVAL STATEMENT

The study protocol was approved by the Ethics Committee of Tongji Medical College, Huazhong University of Science and Technology (No: IORG0003571).

## Supporting information


Figure S1

Figure S2

Figure S3

Figure S4

Table S1

## Data Availability

All data created and/or analyzed during this work included in this article.
