# Supplementary figures and images for: Identification of miR‐30c‐5p as a tumor suppressor by targeting the m6A reader HNRNPA2B1 in ovarian cancer
Source: Cancer Med. 2022 Oct 18;12(4):5055–70. doi: 10.1002/cam4.5246 (PMC9972042; doi:10.1002/cam4.5246)

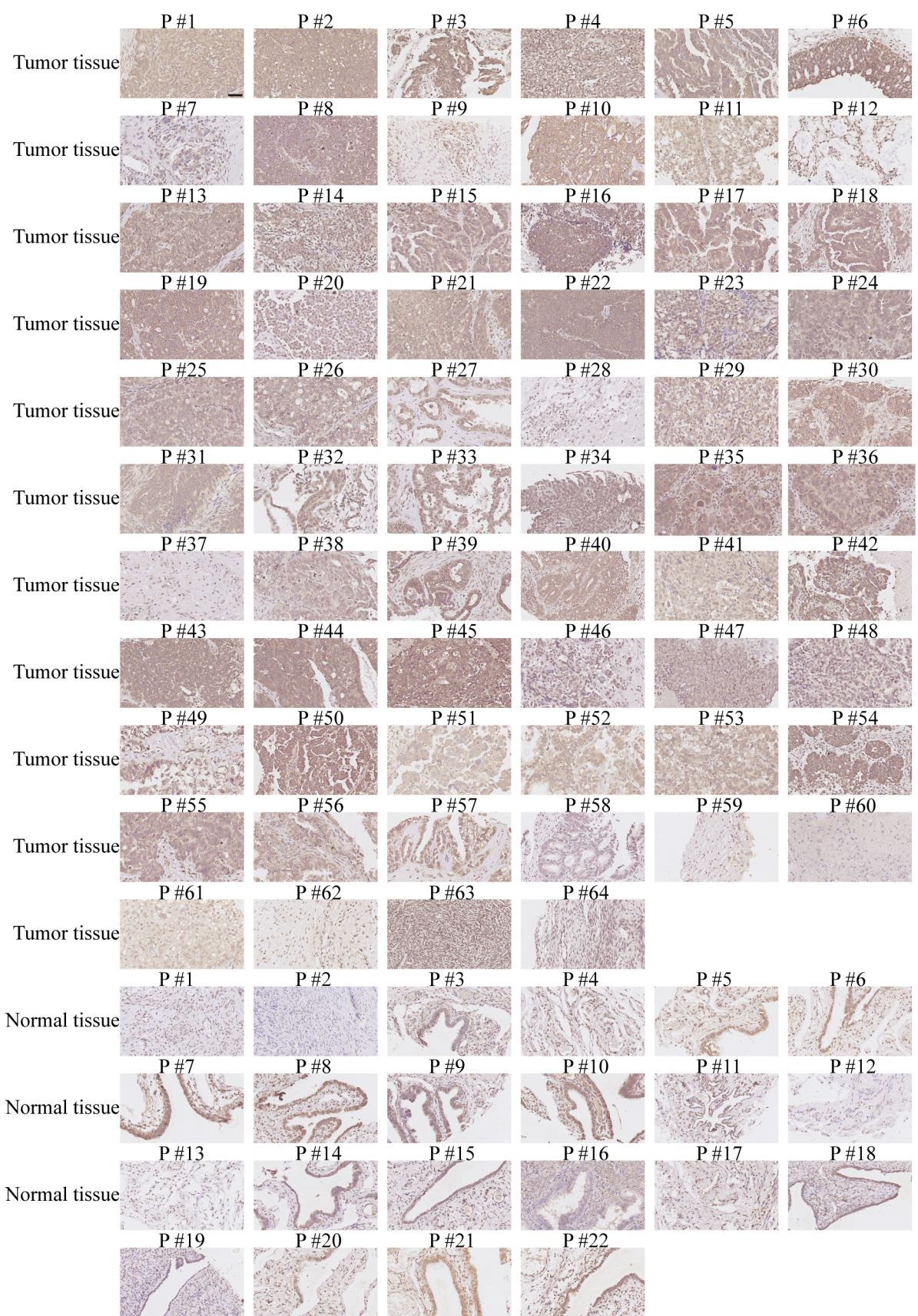

Supplement: Supplementary file 1 — Figure S1 [file CAM4-12-5055-s002.pdf]

a

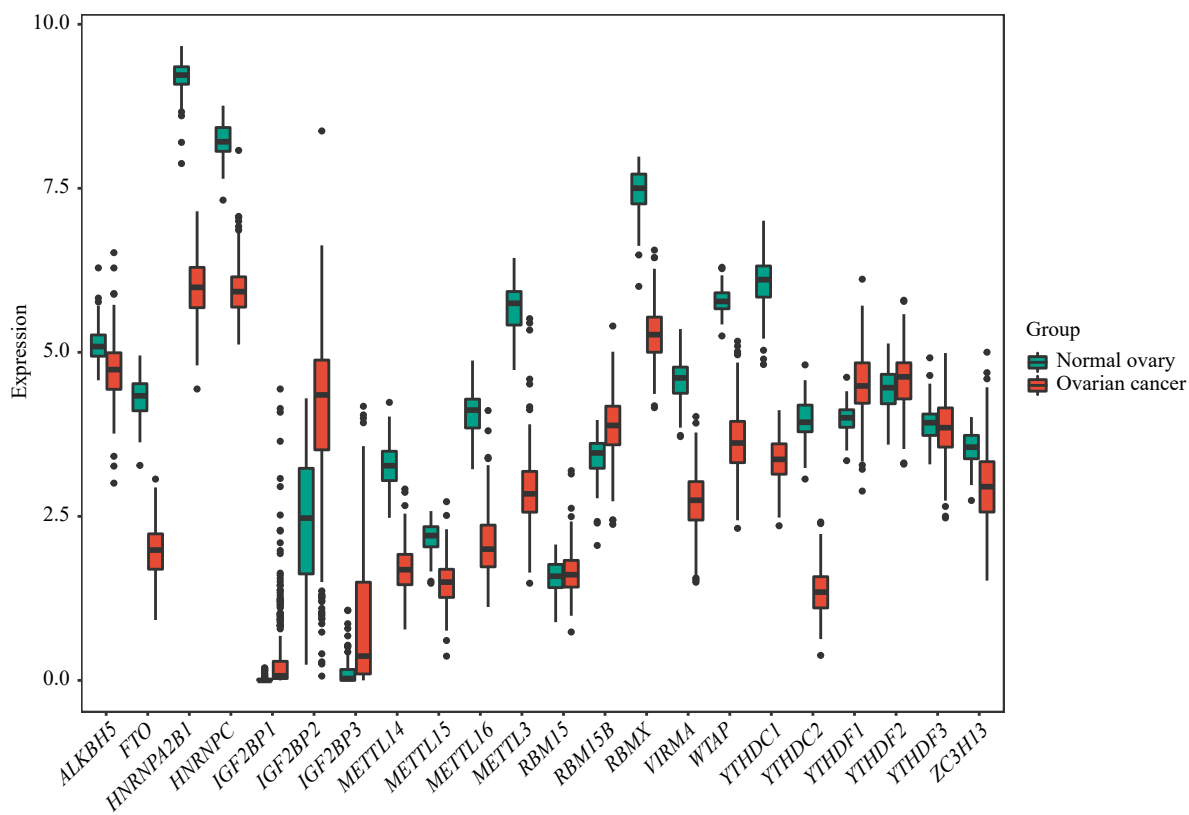

b

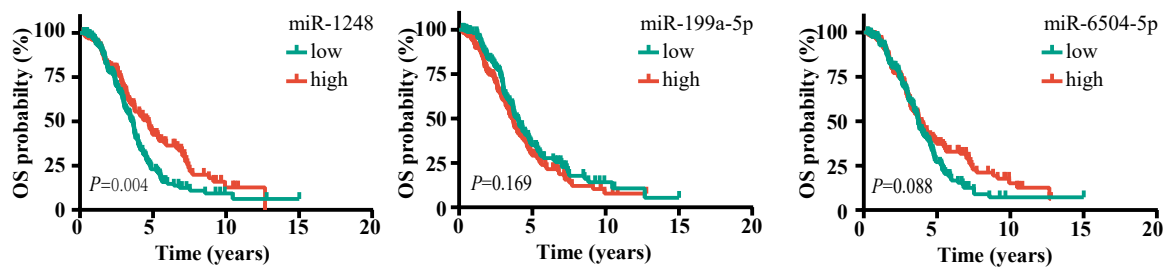

c

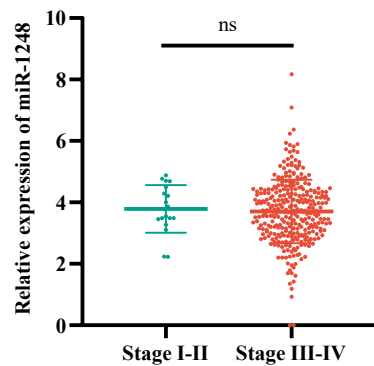

Supplement: Supplementary file 2 — Figure S2 [file CAM4-12-5055-s003.pdf]

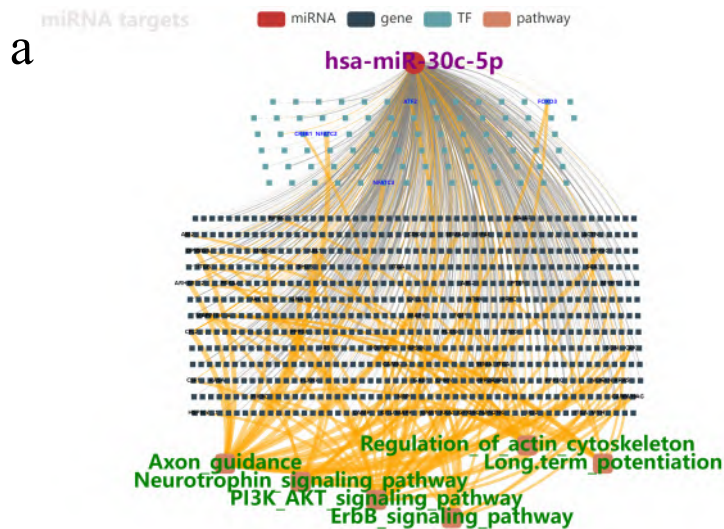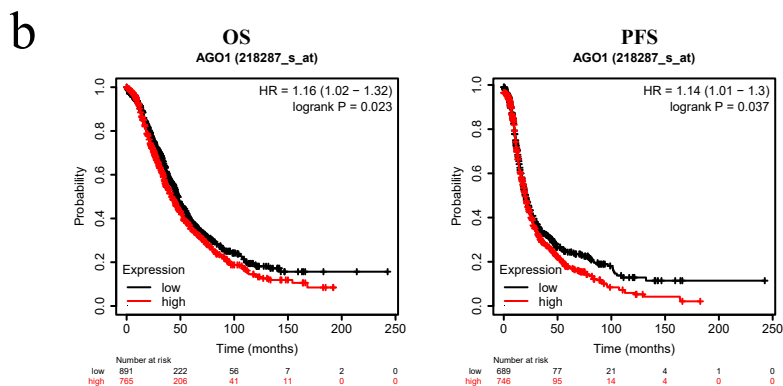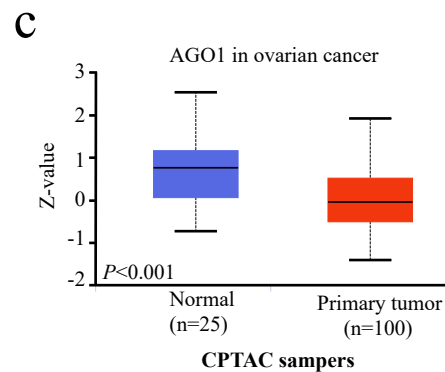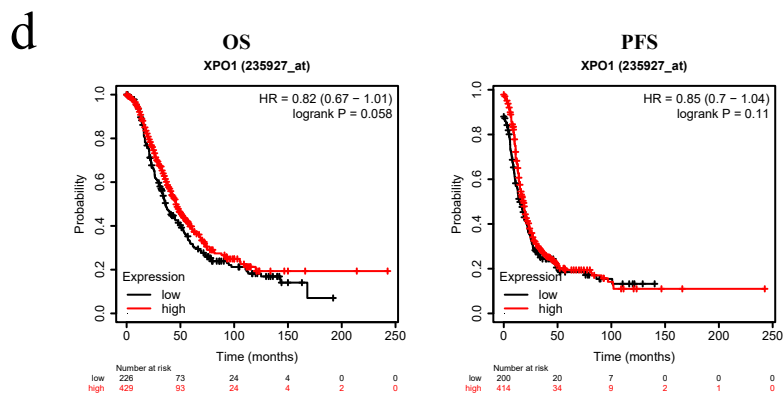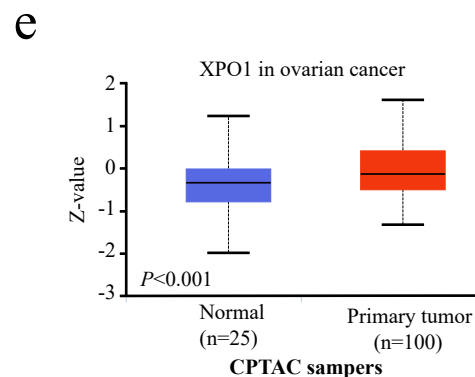

Supplement: Supplementary file 3 — Figure S3 [file CAM4-12-5055-s004.pdf]

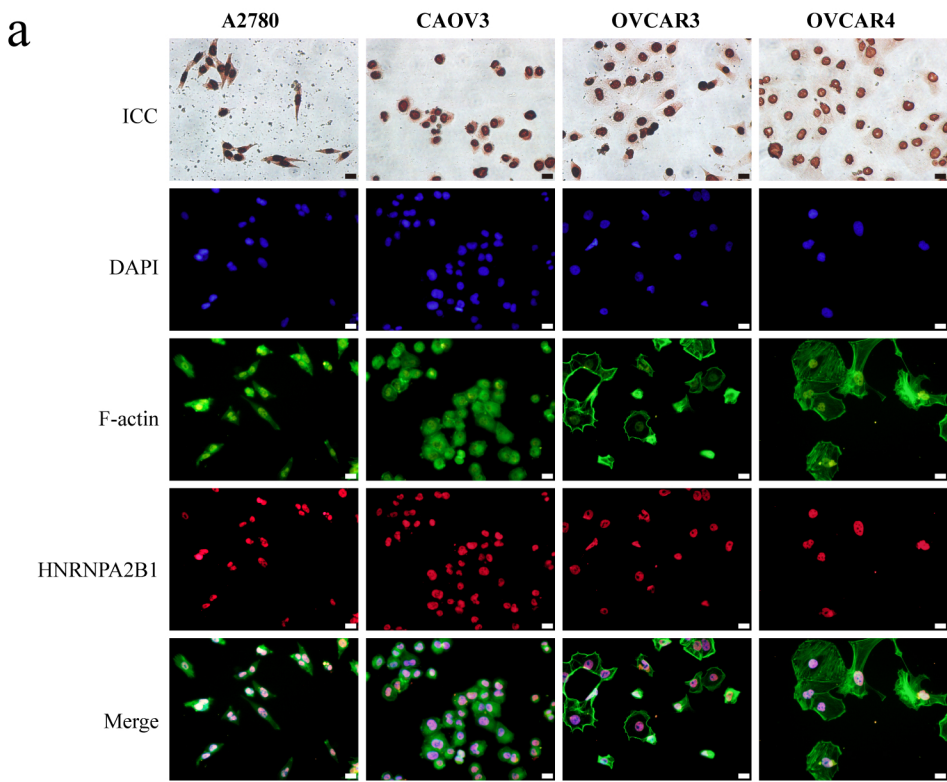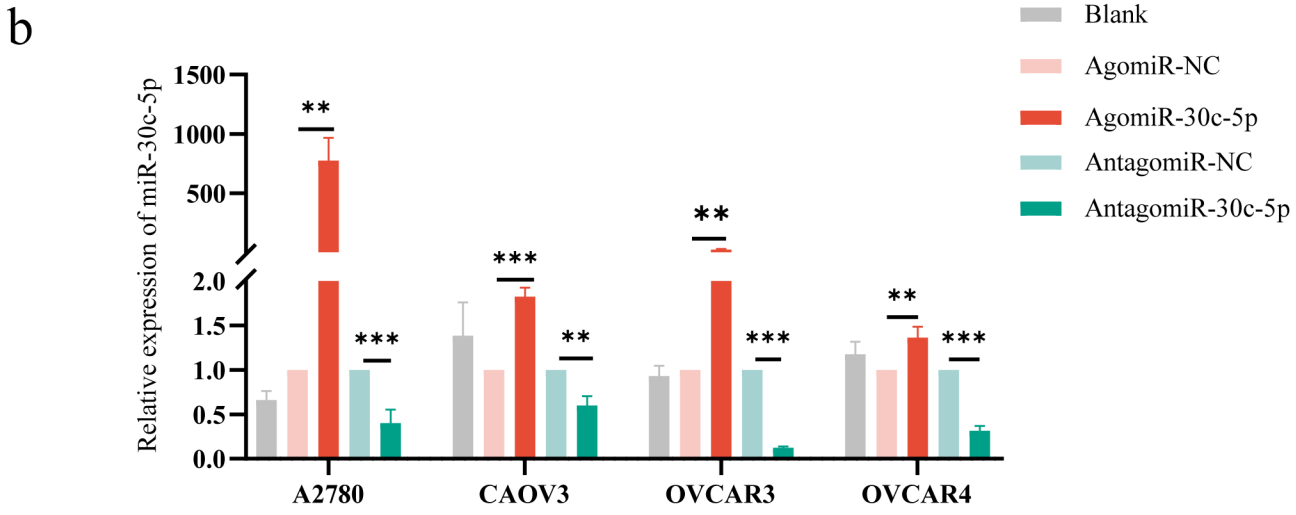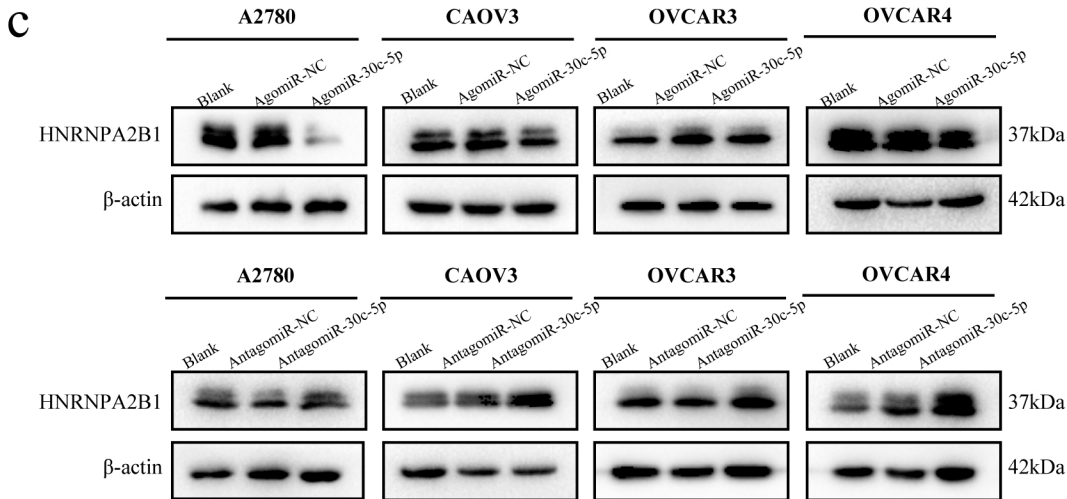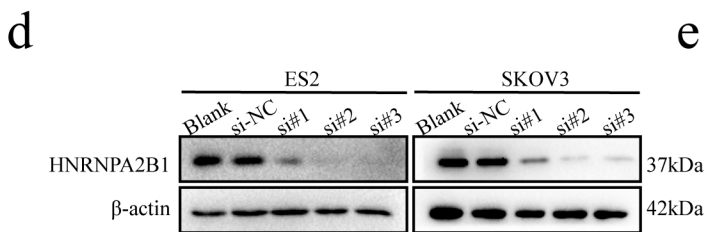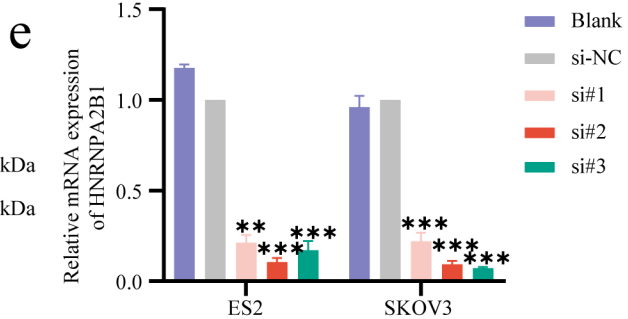

Supplement: Supplementary file 4 — Figure S4 [file CAM4-12-5055-s005.pdf]
