# Supplementary material for: Identification of miR‐30c‐5p as a tumor suppressor by targeting the m6A reader HNRNPA2B1 in ovarian cancer
Source: Cancer Med. 2022 Oct 18;12(4):5055–70. doi: 10.1002/cam4.5246 (PMC9972042; doi:10.1002/cam4.5246)
Supplement: Supplementary file 5 — Table S1 [file CAM4-12-5055-s001.docx]

| Name | Primers |
| --- | --- |
| miR-30c-5p F  miR-30c-5p R | GCGCGTGTAAACATCCTACACT  AGTGCAGGGTCCGAGGTATT |
| U6 F  U6 R | GCTTCGGCAGCACATATACTAAAAT  CGCTTCACGAATTTGCGTGTCAT |
| HNRNPA2B1 F  HNRNPA2B1 R  β-actin F  β-actin R  ZNF12 F  ZNF12 R  ARID1B F  ARID1B R  SACS F  SACS R  LUZP1 F  LUZP1 R  FAM120B F  FAM120B R  CDK19 F  CDK19 R  AFDN F  AFDN R  BRPF1 F  BRPF1 R | ATTGATGGGAGAGTAGTTGAGCC  AATTCCGCCAACAAACAGCTT  CATGTACGTTGCTATCCAGGC  CTCCTTAATGTCACGCACGAT  ACAGGTGTGAAACTCTACAAGTG  CCTCAGGTGGGTCGTGAGA  GCAAGGTGTGAGTGGTTACTG  GGACTGGGACGGCAGATACT  ACAACAACGCGGTTTTCACC  GCCTGATTCATGTGGGCCAA  AGGACCGCCTGGATAAAACTG  TCCTCAATCCGTAGGTCATTTCT  GCACCACCGAAGCAAGTATC  CCACCGCAGATCCAAGATTC  GGATTTGTTTGAGTACGAAGGGT  CTACAAGCCGACATGGATATTCC  ATTTCGACCTGATATGCGAATGC  CAAATCTGCCTTCCCGATCAT  GCCGTGAGGTGATGAGCTATG  TGGTGATGGTTGGAGTCCTTG |
| HNRNPA2B1-si-1-F  HNRNPA2B1-si-1-R  HNRNPA2B1-si-2-F  HNRNPA2B1-si-2-R  HNRNPA2B1-si-3-F  HNRNPA2B1-si-3-R | CAGAAAUACCAUACCAUCAAU  AUUGAUGGUAUGGUAUUUCUG  GAGGUGGUUAUGACAACUATT  UAGUUGUCAUAACCACCUCTT  GCUGCAAGACCUCAUUCAATT  UUGAAUGAGGUCUUGCAGCTT |
